# Effect of Dry Processing of Coconut Oil on the Structure and Physicochemical Properties of Coconut Isolate Proteins

**DOI:** 10.3390/foods13162496

**Published:** 2024-08-08

**Authors:** Xiaoyan Liu, Duwei Yang, Wantong Liu, Jintao Kan, Yufeng Zhang

**Affiliations:** 1 Hainan Engineering Center of Coconut Further Processing, Coconut Research Institute of Chinese Academy of Tropical Agricultural Sciences, Wenchang 571339, China; liusmile18@163.com (X.L.); kanjt@foxmail.com (J.K.); 2 College of Tropical Crops, Yunnan Agricultural University, Puer 665099, China; 15887658450@163.com; 3 College of Food Science and Technol, Huazhong Agricultural University, Wuhan 430070, China; liuwantong0568@163.com

**Keywords:** coconut isolate protein, structure, physicochemical properties, dry processing

## Abstract

The effects of the dry processing of coconut oil on the amino acid composition, molecular weight, secondary structure, solubility, surface hydrophobicity, microstructure, total sulfhydryl and free sulfhydryl content, free amino acid content, thermal properties, and water-holding, oil-holding, foaming, and emulsifying properties of coconut isolate protein were investigated. The results showed that the dry processing altered the amino acid composition of coconut isolate proteins as well as resulted in fewer irregular structural regions and more homogeneous particle sizes, leading to an improvement in the thermal stability of the proteins. SDS-PAGE analysis showed that globular proteins located at ~34 kDa in coconut isolate proteins underwent slight degradation during the dry processing of coconut oil. The dry processing reduced the surface hydrophobicity, total and free sulfhydryl groups, solubility, and free amino acid content of coconut isolate proteins. In addition, the water-holding capacity, oil-holding capacity, and foam stability of coconut isolate proteins were improved to different degrees after the dry processing. Therefore, the development and utilization of copra meal protein is of great significance to increase its added value.

## 1. Introduction

Coconut palm (*Cocos nucifera* L.) is one of the most important woody oil plants and fruit trees in tropical and subtropical regions. Coconut has high economic and nutritional values and is praised as the “tree of life” [1]. In China, coconut resources are mainly grown in Hainan Province. The planting area of Hainan Province is more than 36,000 hm^2^, and the production of coconut fruits exceeded 200 million in 2021. At present, coconut meat has been developed into a series of products, such as desiccated coconut, coconut oil, coconut sugar, coconut milk, and coconut powder [2,3].

Coconut meat is the most exploited part of the coconut fruit, consisting of 37.3% fat, 4.1% protein, and 46.3% water [4]. Coconut oil is the most important product of coconut meat processing, which is usually obtained by “dry-processing” in industry. Dry processing involves the pulverization of coconut meat, which has been dried in the open air and then mechanically extruded to produce coconut oil [5]. Copra meal is a by-product of the oil extraction process and is usually used as animal feed, fertilizer, or discarded. Copra meal has a protein content of about 19% and its amino acid composition is approximately the same as that of fresh coconut meat and desiccated coconut [6,7,8]. Protein from the coconut meat source contains eight amino acids needed by the body, which have a stimulating effect on the body’s metabolism [9]. In addition, it has a variety of benefits such as lowering blood lipids and inhibiting hyperlipidemia [10]. As far as current development is concerned, the protein resources in copra meal have not been well utilized.

With the diversification of modern diets and the improvement in health awareness, the proportion of plant protein beverages and high-protein substitute meal foods in the entire food industry is gradually increasing [11]. Some scholars [12] argued that copra meal protein can be used as an substitute food ingredient, and its addition can help to improve the quality of food, so the development of new food products based on copra meal protein is conducive to the rational use of coconut resources. It is unclear whether the dry processing of coconut oil leads to changes in the structural and functional properties of coconut proteins. However, the final protein nutritional value of coconut products depends on the nature and extent of dry processing. Therefore, it is necessary to understand the changes in protein structure and functional properties before and after oil extraction.

In recent years, most of the domestic and international studies on coconut proteins have focused on modification and emulsion, and little research has been conducted on the changes in the physicochemical properties of proteins before and after oil extraction. Therefore, in the present study, desiccated coconut (coconut meat with water removed) and copra meal (the product remaining after the extraction of coconut oil from desiccated coconut by dry processing) were used as raw materials, and then alkaline extraction and acid precipitation methods were used to extract the proteins from the two coconut meat sources. The changes in the structural as well as functional properties of proteins were analyzed by using Fourier transform infrared spectroscopy, SDS gel electrophoresis, water-holding and emulsifying properties, and so on. The aim of this study was to determine the effect of dry processing on the structural and functional properties of coconut proteins, and the results of the study provide a reference for the application of copra meal proteins in the food industry.

## 2. Experimental Section

### 2.1. Chemicals and Reagents

Desiccated coconut and copra meal were provided by local coconut factory in Wenchang (Hainan, China). Soy isolate protein (SIP), sodium hydroxide (NaOH), hydrochloric acid (HCl), coomassie brilliant blue, sodium dodecyl sulfate (SDS), trometamol, glycine, ethylene diamine tetraacetic acid, 5,5′-Dithiobis-(2-nitrobenzoic acid) (DTNB), methanol, 8-anilino-1-naphthalenesulfonic acid (ANS), ammonium persulphate (AP), urea, *N,N,N′,N′*-tetramethylethylenediamine, acetic acid, and 30% acrylamide were analytical reagent grade and obtained from Wuxi Jingke Chemical Co., Ltd. (Wuxi, Jiangsu, China).

### 2.2. Pre-Treatment of Raw Materials and Protein Extraction

Desiccated coconut and copra meal were crushed and then continuously defatted by the Soxhlet method for 8 h at 45 °C. The defatted desiccated coconut and copra meal were dried in an oven at 45 °C and collected in a desiccator for subsequent tests.

The defatted samples were mixed with 0.1 mol/L NaOH solution at a 1:15 ratio (*w*/*v*) and stirred continuously for 2 h at room temperature. After centrifugation at 3027× *g* for 20 min, the supernatant was collected. The precipitate was subjected to a second alkaline extraction and centrifugation. The supernatants obtained from the two centrifugations were combined and the pH was adjusted to 3.5~4.5 with 2 M HCl to precipitate the proteins. After centrifugation at 3027× *g* for 20 min, protein precipitates were obtained. Finally, the precipitate was loaded into dialysis membranes with a molecular weight cut-off of 3500 Da and dialyzed to remove impurities. The dialyzed precipitate was lyophilized and stored in a desiccator for subsequent experiments. The protein sample obtained from defatted copra meal was named CMP, and the protein sample obtained from defatted desiccated coconut was named DCP.

### 2.3. Proximate Composition and Amino Acid Composition

The moisture, protein, ash, total carbohydrate, and fat content of defatted desiccated coconut, defatted copra meal, DCP, and CMP were measured according to Zhang et al. [1]. The extracted DCP and CMP (20 mg) were acid hydrolyzed by 6 M HCl at 110 °C for 24 h and then derivatized with AccQ-fluor derivatization reagent for 30 min. After that, the amino acid composition of hydrolyzed samples was identified by an automated amino acid analyzer (L-8900, Hitachi Ltd., Tokyo, Japan).

### 2.4. Sodium Dodecylsulphate Polyacrylamine Gel Electrophoresis (SDS-PAGE)

The SDS-PAGE was conducted as previously described by Patil and Benjakul [13]. Briefly, the SDS-PAGE of samples was performed in 5% stacking gel and 15% separating gel using a Thermo Fisher apparatus. Protein samples were mixed with sample buffer (containing Tris-HCl, SDS, bromophenol blue, glycerol, β-mercaptoethanol, and distilled water) at 3:1 volume and boiled. The mixture was cooled to room temperature and centrifuged at 9390× *g* for 3 min. An appropriate amount of supernatant was added to the top sample hole for electrophoresis. After electrophoresis, the gel was stained for 2 h and then decolorized for 12 h.

### 2.5. Fourier Transform Infrared Spectroscopy (FT-IR)

The FT-IR data of protein samples were detected using a FT-IR spectrometer. Protein sample and KBr were mixed well in a mortar in the ratio of 1:100 and pressed into tablets for further determination. Each spectrum was scanned 32 times in the wavenumber range of 4000–600 cm^−1^ at a resolution of 8 cm^−1^. The amide I bands in the sample spectra were baseline-corrected, smoothed, de-convolved, and fitted with second-order derivatives using Peakfit 4.12 software. The relative amount of secondary structure was then obtained by calculating the area of each subpeak.

### 2.6. Thermogravimetric Analysis (TGA) and Differential Scanning Calorimetry (DSC)

A 5 mg sample was placed in triplicate in an aluminum pan. The TGA apparatus was operated with the following parameters: heating rate of 10 °C/min and scan from 35 to 650 °C in an inert nitrogen environment.

### 2.7. Scanning Electron Microscopy (SEM)

A small amount of freeze-dried DCP and CMP samples were uniformly adhered to conductive double-sided tape and then laid flat on a metal sample stage. The sample stage was placed in a vacuum gold plating tray and the samples were vacuum gold-plated. The surface morphology of DCP and CMP was observed by scanning electron microscopy 3200 (CIQTEK Co., Ltd., Hefei, China) under 10 kV voltage.

### 2.8. Sulfhydryl Group (SH) Contents Determination

The determination and calculation of the sulfhydryl group (SH) of protein samples were carried out referred to Deng et al. [14]. An amount of 1 mL protein solution (10 mg/mL) was added to 2 mL buffer solution Ι (for determination of total SH) or buffer solution II (for determination of free SH). An amount of 0.02 mL of Ellman’s reagent (0.2 g DTNB in 50 mL of buffer I) was added and incubated for 5 min at room temperature. After that, the absorbance of mixture at 412 nm was measured. SH content was calculated as follows:

(1) $$SH\;content\;(\mu mol/g)=\frac{73.53\times A\times D}{C}$$

where A is the absorbance of the sample solution at 412 nm; 73.53 = 10^6^/(1.36 × 10^4^); 1.36 × 10^4^ is the molar extinction coefficient; D is the dilution factor of the sample; and C is the original protein concentration.

### 2.9. Free Amino Content

The OPA reagent was prepared using a method of Zhang et al. [15]. An amount of 400 μL protein solution (10 mg/mL) was mixed with 3 mL OPA reagent. The absorbance was measured at 340 nm after incubation for 3 min at 25 °C. Finally, the free amino acid content of the protein samples was calculated by plotting a standard curve based on serine.

### 2.10. Determination of Surface Hydrophobicity (H_0_)

The H_0_ of SIP, DCP, and CMP was measured to refer to the method of Pi et al. [16] with slight modifications. Six different concentration (0.05, 0.1, 0.2, 0.3, 0.4, and 0.5 mg/mL) protein solutions were prepared by dissolving the sample in 0.01 mol/L phosphate buffer solution (pH 7.0). An amount of 20 μL of the fluorescent probe ANS (8 mmol/L) was added to 2 mL protein solution and mixed evenly. After incubation for 15 min protected from light at room temperature, fluorescence intensity was detected with a fluorescence spectrophotometer. The excitation wavelength was set to 390 nm with a slit correction of 5 nm, and the emission wavelength was set to 470 nm with a slit correction of 5 nm. By plotting the fluorescence intensity against sample concentration, the slope of the curve represents the surface hydrophobicity index (H_0_) of the sample.

### 2.11. Protein Solubility (PS)

The PS was studied and calculated with reference to Naik et al. [17] with some modifications. An aqueous protein solution of 5 g/L was obtained by dispersing 500 mg of protein sample in 100 mL of distilled water. The solution was stirred with a magnetic stirrer for 1 h. Subsequently, the solution was centrifuged at 1728× *g* for 20 min to obtain the supernatant. The supernatant was filtered into a 100 mL volumetric flask and fixed with distilled water. The supernatant protein content was estimated by the Bradford method using bovine serum protein as a standard. Protein solubility was calculated by the following equation:

(2) $$PS\;(\%)=\frac{C_{1}}{C_{2}}\times 100\%$$

Notably, C_1_ is the protein content of supernatant; and C_2_ is the total protein content of the protein sample.

### 2.12. Measurement of Water-Holding Capacity (WHC) and Fat Absorption Capacity (FAC)

The WHC and FAC of protein samples were evaluated based on the method from Deng et al. [14] with some modifications. An amount of 1 g of the protein sample and 10 mL distilled water or soybean oil were placed in a centrifuge tube and vortexed for 30 s. After standing at room temperature for 30 min, the mixtures were centrifuged at 1728× *g* for 25 min. The supernatant was discarded and the precipitate was weighed. WHC and FAC were expressed as grams of water or oil absorbed per gram of protein sample.

### 2.13. Foaming Properties

The foaming capacity (FC) and foaming stability (FS) of protein samples were measured according to Pi et al. [16] with minor modifications. Briefly, 100 mL protein solutions (10 mg/mL) were homogenized at 9390× *g* for 2 min and immediately transferred to a dry and clean measuring cylinder. The total volume of foam was recorded at 0 min and after 30 min of standing. Referring to the calculation method provided by Pi et al. [16], the formula of FC and FS is as follows:

(3) $$FC\;(\%)=\frac{V_{1}}{100}\times 100\%$$

(4) $$FS\;(\%)=\frac{V_{2}}{V_{1}}\times 100\%$$

In the above equation, V_1_ represents the foam volume at 0 min. After 30 min, V_2_ is the foam volume; 100 is the initial volume of the protein solutions.

### 2.14. Emulsification Properties

The determination methods and calculation formulas of emulsion activity index (EAI) and emulsion stability index (ESI) of protein samples refer to Wu et al. [10]. The protein sample was dispersed in distilled water to obtain 1% (*w*/*w*) solution. An amount of 10 mL 1% solution was added to 20 mL of soybean oil and vortexed briefly. Subsequently, the mixture was homogenized at 9390× *g* for 3 min and then left to stand. An amount of 100 μL of the solution was pipetted from the bottom of the emulsion and mixed with 5 mL of 0.1% SDS at 0 min and 30 min of resting. The absorbance values were determined at 500 nm. The calculation formula for EAI and ESI was as follows:

(5) $$EAI\;(m^{2}/g)=\frac{\left({2\times 2.2303\times A}_{0}\times N\right)}{\left(c\times \Phi \times L\times 10,000\right)}$$

where A_0_ is the absorbance value at 0 min; N is the dilution ratio; c is the protein concentration (g/mL); Φ is the oil phase volume fraction (2/3); L is the optical path length (1 cm); 2 is the average scattering coefficient; and 2.303 is the scale coefficient.

(6) $$ESI\;(min)=\frac{\left(A_{0}\times ∆t\right)}{\left(A_{0}-A_{30}\right)}$$

where A_30_ is the absorbance value at 30 min; Δt is the time difference (min).

### 2.15. Statistical Analysis

In this study, all physico-chemical measurements of DCP and CMP were performed in triplicate. One-way analysis of variance (ANOVA) was performed using Duncan’s test in SPSS software version 26.0 (SPSS Inc., Chicago, IL, USA) to analyze significant differences between means (*p* < 0.05).

## 3. Results and Discussions

### 3.1. Analysis of Proximate Composition and Amino Acid Composition

Chemical compositions of defatted desiccated coconut, defatted copra meal, DCP, and CMP are shown in Table 1. As observed, protein was the major composition of raw materials and protein samples. In addition, the ingredients and samples contain some carbohydrates. Coconut meat is rich in carbohydrates, mainly dietary fiber [18]. During the protein extraction process, some dietary fiber and protein were soluble in NaOH solution. However, due to structural and compositional differences, crude fiber does not possess the unique structural and functional properties of proteins and has no significant effect on subsequent experiments.

The essential amino acid profile that the Food and Agriculture Organization/World Health Organization (FAO/WHO) recommends for adults and children was compared to the amino acid compositions (mg/g protein) of SIP (soy isolate protein), DCP (isolated proteins from defatted desiccated coconut), and CMP (isolated proteins from defatted copra meal), which are given in Table 2. It can be observed that the three proteins had the same amino acid composition but different contents. The essential amino acids (EAA) of DCP and CMP accounted for 37.87% and 37.21% of the total amino acids, respectively, higher than that of SIP (35.44%). The content of EAA in DCP and CMP reached the minimum requirement of 27.7% set by FAO/WHO. The essential amino acid content of CMP decreased compared to DCP, which suggests that the dry processing caused a slight loss of essential amino acids in coconut proteins. The imbalance in the composition of essential amino acids was a major constraint to the use of plant proteins in the food industry [14].

The lysine levels in DCP and CMP were slightly lower than the FAO/WHO recommendations (2007), as seen in Table 2. However, the levels of other essential amino acids were higher than the FAO/WHO recommended intakes. Therefore, CMP can still be an important source of essential amino acids for the food industry.

The most prevalent amino acids in DCP and CMP were glutamic acid (18.16–19.78%) and arginine (11.33–11.20%), which can be considered dominant content amino acids because of their increased abundance in comparison to other amino acids. In addition, except for phenylalanine, lysine, and histidine in CMP, which were slightly higher than that in DCP, the levels of other essential amino acids in CMP showed a decreasing trend. The composition of amino acids may influence the functional properties of protein samples. The contents of HAAs (Ala, Val, Ile, Leu, Phe, Pro, and Met) for DCP and CMP were 37.971% and 36.854%, respectively. Research has indicated that a high proportion (27.70%) of hydrophobic amino acids (HAAs) in proteins improves their stability and facilitates lipid dispersion in emulsions [19]. Although the dry processing caused a decrease in the HAA content of CMP compared to DCP, CMP may still be highly stable in emulsions.

### 3.2. SDS-PAGE

**Figure 1:**
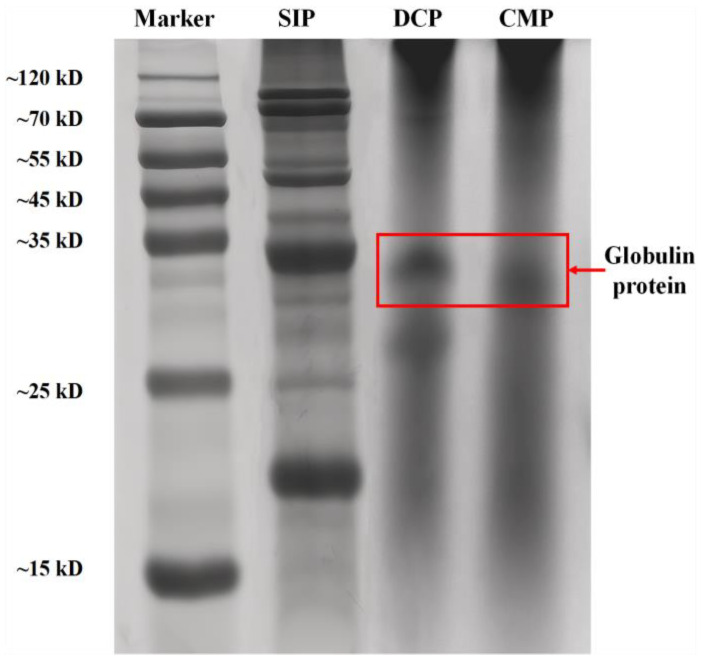
SDS-PAGE analysis of SIP, DCP, and CMP. SIP: soy isolate protein; DCP: isolate protein from desiccated coconut; CMP: isolate protein from copra meal.

SDS-PAGE profiles can characterize the relative abundance and integrity of protein fractions as a whole. In order to further investigate the extent to which dry processing affects the protein structure of desiccated coconut, SDS-PAGE of DCP and CMP was determined under non-reducing conditions. Figure 1 shows that in the DCP, two prominent bands are present at high abundance at ~27 kDa and ~34 kDa, respectively, while most of the other bands are present at low abundance and low resolution. CMP shows a significant band only at ~32 kDa. Meanwhile, some shadows were piled up at the top in both DCP and CMP lanes, implying that there were substances with higher molecular mass in both proteins that failed to enter the electrophoretic bands.

Based on previous reports [13], it is known that globulin is the most abundant protein in coconut meat and its electrophoretic band is located around 35 kDa. Therefore, it can be inferred that the bands located at ~34 kDa in DCP and CMP are produced by globulin. Further observation revealed that the molecular weight of globulin in CMP was slightly lower compared to that of DCP, which might be due to the partial degradation of globulin during the dry processing.

### 3.3. Fourier-Transform Infrared Spectroscopy (FTIR)

FTIR spectroscopy has become a useful tool for analyzing the secondary structure of proteins because it can reflect the amide bonding vibrations present in proteins. In the case of proteins, the spatial arrangement is predominantly reflected in the amide I band (1600–1700 cm^−1^), the amide II band (1530–1550 cm^−1^), and the amide III band (1260–1300 cm^−1^). Interestingly, a study has proposed that the amide I band exhibits the strongest correlation with a protein’s secondary structure when compared to the amide II and III bands [20].

**Figure 2:**
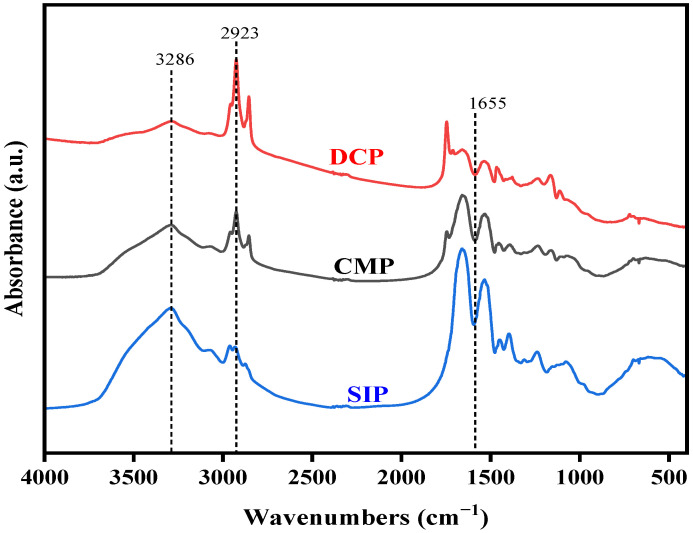
FTIR spectra of SIP, DCP, and CMP in 4000–400 cm^−1^ regions. SIP: soy isolate protein; DCP: isolate protein from desiccated coconut; CMP: isolate protein from copra meal.

The FTIR spectra in Figure 2 illustrate the characteristic frequencies of the amide A band, which arises from O-H and N-H stretching vibrations and typically falls within the range of 3500–3000 cm^−1^. This band provides hydrogen atoms for interactions with other functional groups. Comparing the FTIR spectra of DCP and CMP, the amide A band peak of CMP is significantly narrower than that of DCP, but the intensity of the absorption peak was higher, which result supports the existence of intramolecular hydrogen bonding changes. Furthermore, C-H stretching vibrations corresponding to hydrophobic regions within the protein structure were usually observed in the range of 2800–3000 cm^−1^ [21]. The peaks at 2923 cm^−1^ of DCP and CMP were caused by the asymmetric stretching vibration of CH_2_ group in the fatty side chain. The higher intensity of the CH_2_ group peak indicates that the dry processing affects the hydrophobic domain of the protein. The main peaks appearing in the amide I region were due to the stretching vibrations of the C=O and N=H groups. The characteristic region of β-sheets occurs in the range of 1600–1640 cm^−1^, random coils between 1640 and 1650 cm^−1^, α-helices in the range of 1650–1660 cm^−1^, and β-turns within 1660–1700 cm^−1^. Table 3 displays the contents of α-helix, β-sheet, β-turn, and random coil in SIP, DCP, and CMP based on the simulated peak area of the amide I band.

The α-helix is ordered and susceptible to conformational changes. β-sheet and β-turn structures are relatively stretchable and ordered. In contrast, random coils are considered to be disordered [22]. The reduction in β-sheet structure and random coil structure in CMP suggests that dry processing is more likely to disrupt both structures. Furthermore, the proportion of α-helix and β-sheet structures implies a degree of protein compactness, reflecting the abundance of hydrogen bonds between protein molecules [23]. Table 3 shows that DCP has the highest percentage of α-helix and β-sheet structures, indicating that DCP has a stronger hydrogen bond interaction compared to other samples.

### 3.4. Thermal Properties

**Figure 3:**
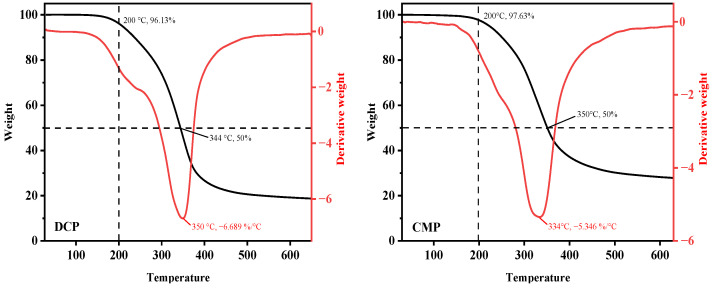
TGA and DTG curves of DCP and CMP. DCP: isolate protein from desiccated coconut; CMP: isolate protein from copra meal.

As can be seen from Figure 3, the degradation of DCP and CMP can be divided into different stages. The first stage mainly occurs in the range of 30–200 °C with weight losses of 3.87% and 2.37%, respectively. The changes in the first stage may be caused by removing free and bound water from the protein samples. The second stage, also known as the “rapid mass loss stage”, occurs in the range of 200 to 450 °C. Mo et al. [24] suggest that the phase is caused by protein degradation. Protein degradation includes the breaking of inter- and intramolecular hydrogen and electrostatic bonds, the breaking of protein side chains, and the breaking of weak C-N, C(O)-NH, C(O)-NH_2_, and NH_2_ bonds. It can be observed that the rate of weight change of both DCP and CMP showed a clear trend of decreasing and then increasing from Figure 3. In addition, DCP and CMP lost 50% of their weight at 344 and 350 °C, respectively. When the temperature continued to rise to 650 °C, the final product (residual carbon) was formed, which was the last stage of protein mass change. A comparison of the DTG curves of DCP and CMP reveals that the rate of change of CMP is much lower than that of DCP in the ranges of 132–248 °C and 318–407 °C.

Therefore, based on the combined consideration of loss rate and temperature change, it can be inferred that CMP exhibits superior heat resistance compared to DCP, which significantly indicates that the thermal stability of the proteins subjected to dry processing has been improved. The improvement may be attributed to the fact that the dry processing enhanced the intermolecular interactions between the protein molecules, which led to the aggregation of the originally disordered polypeptide chains within the protein and the formation of a more organized structure, thus enhancing the thermal stability of the desiccated coconut protein [25].

### 3.5. SEM

**Figure 4:**
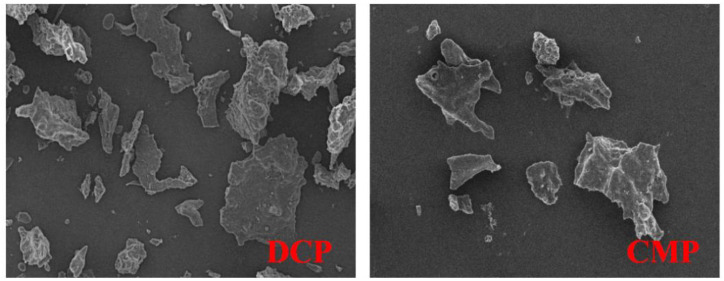
SEM images of DCP and CMP. Magnification: 500×. DCP: isolate protein from desiccated coconut; CMP: isolate protein from copra meal.

As shown in Figure 4, DCP and CMP were freeze-dried and their structural features were observed by SEM. It can be observed that at the same magnification, although both DCP and CMP showed a lamellar structure, their sizes were obviously different. There were fewer small fragments in the SEM image of CMP compared to DCP. Combined with the results of SDS analysis, it can be hypothesized that the proteins in copra were fractured as well as reorganized with each other during the dry processing, resulting in changes in the microstructures of the proteins.

### 3.6. Analysis of Sulfhydryl (SH) Content

SH is a crucial active group in protein molecules that has an important effect on protein function. This is due to the fact that the SH and disulfide (S-S) exchange reaction has the ability to encourage protein polymerization. This type of polymerization produces a highly viscoelastic layer that prevents protein aggregation in addition to allowing the protein to adsorb onto the interface irreversibly [20].

**Figure 5:**
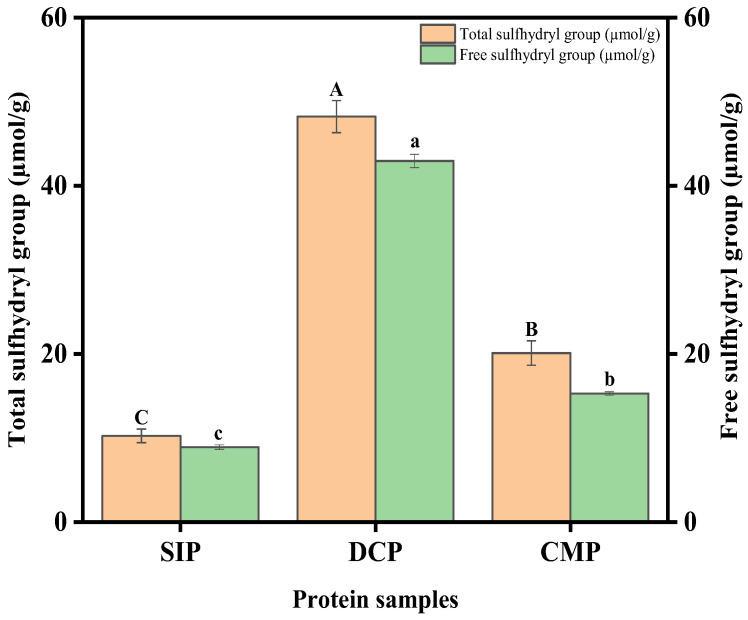
Total sulfhydryl and free sulfhydryl contents of SIP, DCP, and CMP. SIP: soy isolate protein; DCP: isolate protein from desiccated coconut; CMP: isolate protein from copra meal; A–C and a–c: different letters on the bar mean significant difference (*p* < 0.05).

Figure 5 shows the total and free SH content of DCP and CMP, as well as the SH content of SIP. The concept of total SH defines the amount of all sulfhydryl groups present in the protein, including those that are surface-exposed and those that hide within the protein network. It can be seen from Figure 5 that the total and free SH content of DCP and CMP was much higher than SIP. However, dry processing decreased the content of total and free SH of coconut meat from 48.22 to 20.1 μmol/g and 42.95 to 15.28 μmol/g (*p* < 0.05), respectively. Li et al. [26] proposed that the loss of sulfhydryl groups may be due to the formation of disulfide bonds within or between peptides. During dry processing, the mechanical shearing caused by extrusion resulted in a decrease in the surface hydrophobicity of coconut proteins. The reduction in intermolecular force due to the decline in protein surface hydrophobicity will promote the interaction between free sulfhydryl groups to form disulfide bonds. Wang et al. [27] performed extrusion experiments on whey protein isolate and casein. The results showed that the SH content of extruded whey isolate and casein was significantly lower than that of unsqueezed protein.

### 3.7. Analysis of Free Amino Content

**Figure 6:**
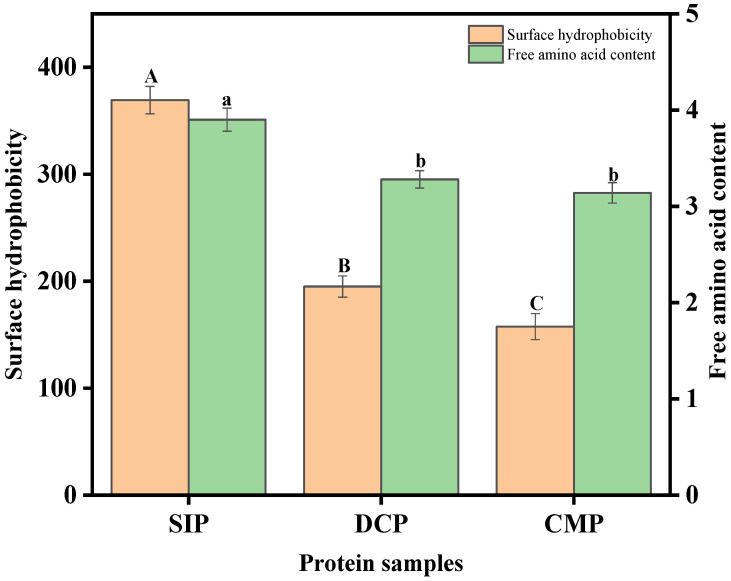
Surface hydrophobicity (*H*_0_) and free amino acid contents of SIP, DCP, and CMP. SIP: soy isolate protein; DCP: isolate protein from desiccated coconut; CMP: isolate protein from copra meal; A–C and a–b: different letters on the bar mean significant difference (*p* < 0.05).

The effect of dry processing on the free amino acid content of coconut protein is depicted in Figure 6. The content of free amino acids of proteins obtained from desiccated coconut (DCP) and copra meal (CMP) was significantly lower than soybean isolate protein (SIP). The free amino acid content of CMP was slightly lower by 0.14% as compared to DCP, which may be attributed to the conditions during dry processing that caused the cross-linking of the protein molecular chains, thus depleting the free amino acids in the system. In addition, based on the results of Sante-Lhoutellier et al. [28], it was reasonable to speculate that the protein structure was unfolded by heat during processing, and the amino side chains buried inside the molecule were exposed and quickly oxidized to generate carbonyl and other groups, leading to changes in the free amino acid content.

### 3.8. Analysis of Surface Hydrophobicity

Maintaining the tertiary structure of proteins is primarily due to the interaction of hydrophobic groups. Therefore, changes in surface hydrophobicity may be a reflection of the structural stability and conformational changes of proteins, which are closely related to the functional properties of proteins. The effect of dry processing on surface hydrophobicity of coconut meat protein is displayed in Figure 6. Dry processing significantly reduced the surface hydrophobicity of coconut proteins. The surface hydrophobicity of CMP was significantly lower than that of DCP. Jiang et al. [29] believed that the decrease in surface hydrophobicity was related to the number of hydrophobic amino acid residues. From the results of amino acid composition in this study, the content of hydrophobic amino acids in CMP was lower than that in DCP, which might be the reason for the decrease in protein surface hydrophobicity.

### 3.9. Functional Characteristics

The functional properties of proteins play a crucial role in how they behave in various food systems during processing, storage, preparation, and consumption. Key properties that are of interest prior to their use in food applications include water and oil absorption capacities, solubility, foaming properties, and emulsifying properties. A comparison of these functional properties was conducted for different types of coconut cakes based on their protein content.

#### 3.9.1. Analysis of Solubility

**Figure 7:**
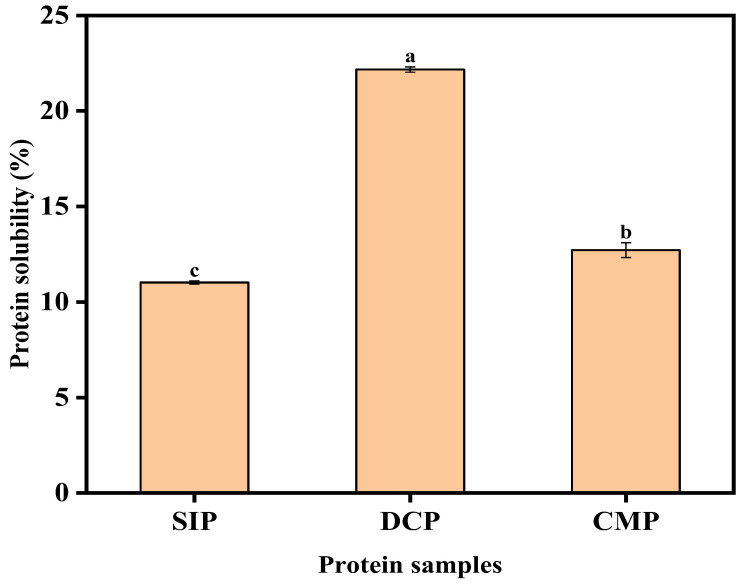
Protein solubility profile of SIP, DCP, and CMP. SIP: soy isolate protein; DCP: isolate protein from desiccated coconut; CMP: isolate protein from copra meal; a–c: different letters on the bar mean significant difference (*p* < 0.05).

The solubility profile of protein is a valuable tool for determining the compatibility of the protein with various food or beverage applications. The protein solubility profiles of SIP, DCP, and CMP are shown in Figure 7. From Figure 7, it can be clearly seen that the solubility of CMP was significantly lower than that of DCP. The reason for this phenomenon may be that the dry processing enhances the interactions between proteins, leading to protein aggregation and precipitation, and reduces the chance of interaction between proteins and water, which further leads to the reduction in the solubility of proteins. Although the solubility of CMP was lower than that of DCP, it was better than that of SIP. In addition, comparing the contents of secondary structures of DCP and CMP with the results of FTIR, it can be found that the content of random coil in CMP was lower than that of DCP, and the content of α-helix structures is higher than that of DCP. Lyu et al. [30] pointed out that the decrease in random content and the increase in α-helix content represent the more stable structure of the protein molecule.

#### 3.9.2. Water-Holding Capacity (WHC) and Fat-Holding Capacity (OHC)

**Figure 8:**
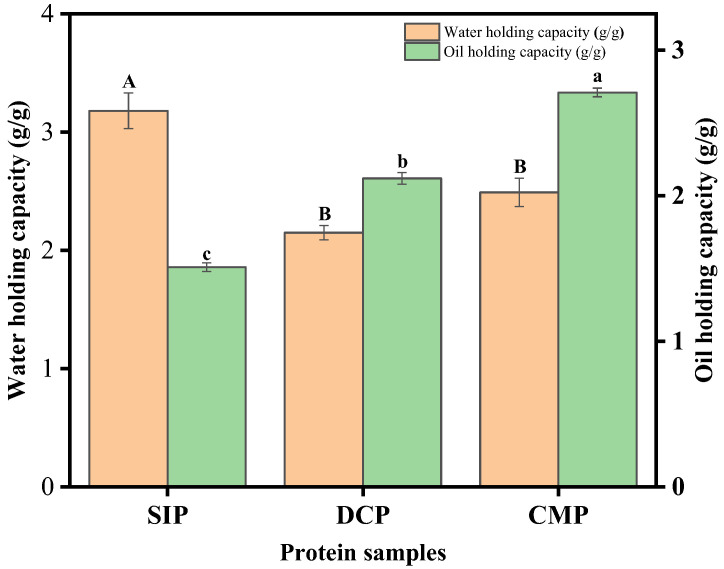
Water-holding capacity (WHC) and fat-holding capacity (OHC) of SIP, DCP, and CMP. SIP: soy isolate protein; DCP: isolate protein from desiccated coconut; CMP: isolate protein from copra meal; A–B and a–c: different letters on the bar mean significant difference (*p* < 0.05).

The water- and oil-holding capacity of proteins refers to the ability of proteins to stabilize water and oil, which is important for practical applications related to proteins in the food industry [31]. The water holdings of SIP, DCP, and CMP are shown in Figure 8. It can be clearly observed that the water-holding capacity of DCP (2.15 ± 0.06 g/g) and CMP (2.49 ± 0.12 g/g) was significantly lower than that of SIP (3.18 ± 0.35 g/g). However, the dry processing resulted in a slight increase in the water-holding capacity of CMP. The water-binding capacity of proteins is influenced by a variety of factors, including structural features, such as protein conformation, hydrophilicity, and hydrophilic balance of amino acids, as well as environmental parameters, such as pH, ionic strength, and temperature. Certain functional properties of proteins, such as water retention and solubility, can be attributed to favorable protein–water interactions. Conversely, other functional properties such as foaming and emulsification stem from unfavorable protein–water interactions. In addition, protein polymer–water interactions are reflected in other key properties such as viscosity, gelation, and coagulation, which are critical in determining the texture, stability, and overall quality of food products [17].

The main factors affecting the strength of oil retention include the structural characteristics of proteins and the participation of non-covalent bonds. The interaction between proteins and lipids is mainly dependent on the interaction of non-polar bonds (such as hydrophobic interaction). This interaction plays a crucial role in proteins acting as emulsifiers to maintain the stability of the oil. Based on the information given in Figure 8, it can be clearly observed that CMP (2.71 ± 0.08 g/g) has the best oil-holding properties, followed by DCP (2.12 ± 0.03 g/g). In general, both DCP and CMP have higher oil-holding properties than SIP (1.51 ± 0.03 g/g). Therefore, it can be assumed that the dry processing improves the oil-holding capacity of proteins.

#### 3.9.3. Foaming Properties

**Figure 9:**
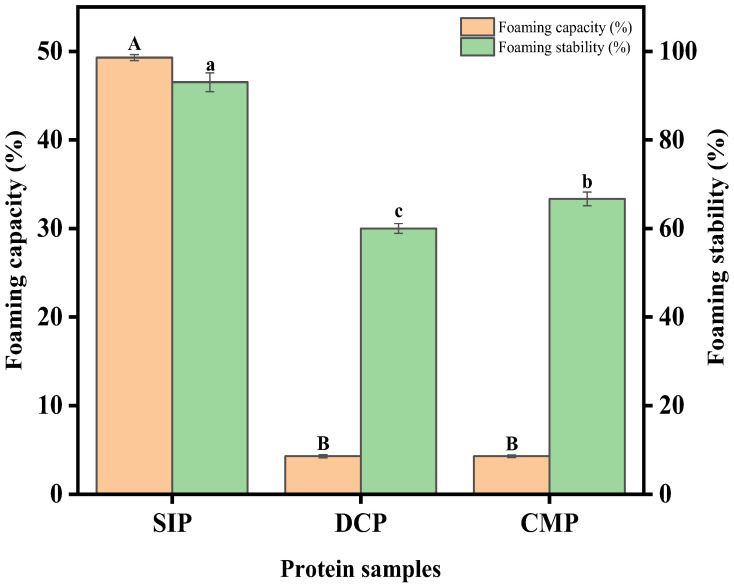
Foaming capacity (FC) and foaming stability (FS) of SIP, DCP, and CMP. SIP: soy isolate protein; DCP: isolate protein from desiccated coconut; CMP: isolate protein from copra meal; A–B and a–c: different letters on the bar mean significant difference (*p* < 0.05).

Figure 9 shows the foaming capacity (FC) and foaming stability (FS) of the SIP, DCP, and CMP. Figure 9 illustrates that the FC and FS of SIP were greater than those of DCP and CMP, although the FC of DCP and CMP do not differ significantly. Du et al. [32] concluded that more hydrophobic groups and hydrophobic regions exposed to the surface cause an increase in the degree of protein aggregation, which reduces the protein activity and leads to a corresponding decrease in the FS value. In a word, high H_0_ values also corresponded to higher capacity and stability of the foam due to the exposure of hydrophobic groups enabling rapid adsorption at the air–water interface [33]. Tian et al. [34] suggested that the SH content is related to the foaming ability of proteins, and the lower the SH content, the more unfavorable it is to the foaming ability of proteins, resulting in a gradual decrease in the foam of the protein solution system. However, the results of the present experiments do not follow this conclusion. For example, the FS of CMP is higher than that of DCP. In summary, it can be speculated that the FC and FS of proteins may depend on the combined effect of changes in their multiple structures and properties (surface hydrophobicity, sulfhydryl content, solubility, etc.) [35].

#### 3.9.4. Emulsifying Properties

**Figure 10:**
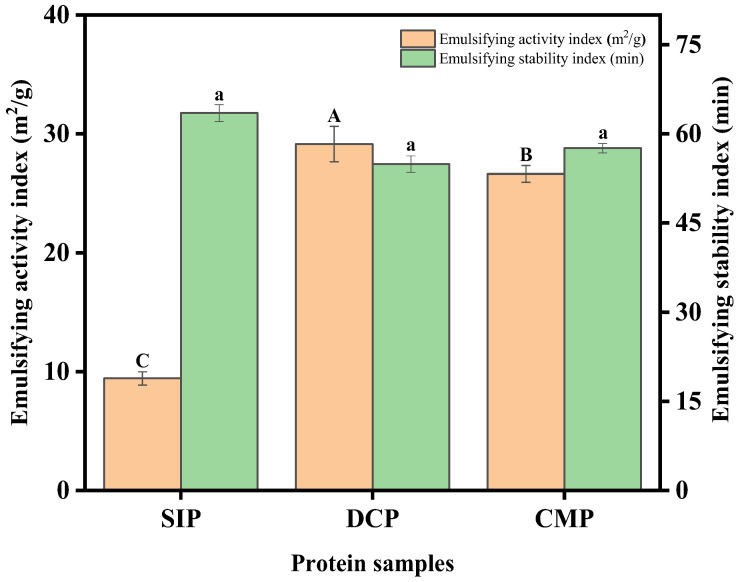
Emulsifying activity index (EAI) and emulsifying stability index (ESI) of SIP, DCP, and CMP. SIP: soy isolate protein; DCP: isolate protein from desiccated coconut; CMP: isolate protein from copra meal; A–C and a: different letters on the bar mean significant difference (*p* < 0.05).

The emulsion activity index (EAI) and emulsion stability index (ESI) are commonly used to evaluate the emulsification properties of proteins. The EAI represents the maximum surface area produced per gram of protein, whereas the ESI indicates the ability to maintain the structure of the emulsion in the face of variations [32]. It can be observed from Figure 10 that the highest EAI was DCP (29.14 m^2^/g), followed by the CMP (26.63 m^2^/g). Emulsification capacity is related to protein solubility, surface charge, surface hydrophobicity, and secondary structure. Ma et al. [36] suggested that the unfolding and degradation of protein structure leads to increased conformational flexibility and exposure of hydrophobic amino acids, which promotes rapid adsorption of proteins at the oil–water interface and reduces interfacial tension, thereby enhancing emulsification capacity.

From the analysis results of 3.1 and 3.8, it can be seen that dry processing leads to lower hydrophobic amino acid and surface hydrophobicity of CMP than DCP, which may be the reason why the EAI of DCP is higher than CMP. Although the EAI of SIP was lower, there was no significant difference in emulsion stability between SIP, CMP, and DCP in terms of the emulsion stability index (ESI). In conclusion, DCP and CMP have good emulsification properties and can be used as ingredients in emulsified foods (mayonnaise, ice cream, and milk).

## 4. Conclusions

The results of this study showed that dry processing changed the physicochemical and functional properties of copra meal isolate protein. The dry processing led to a decrease in the essential amino acid content of CMP compared to DCP, but it was still higher than that of SIP. Based on SDS, it is known that dry processing resulted in degradation of the coconut isolate protein bands located at ~34 kDa. FTIR analysis showed that dry processing led to changes in the secondary and tertiary structure of the protein (increase in α-helix and β-turn content and decrease in β-sheet and random content), which in turn caused a decrease in the surface hydrophobicity, solubility, free amino group, total sulfhydryl groups, and free sulfhydryl groups of the protein. Thermal analysis showed higher thermal stability of the proteins after dry processing. In addition, SEM showed that the dry processing produced the appearance of small pieces of more uniform size. After dry processing, the water-holding and oil-stabilizing properties of CMP were improved, and the foaming properties were not significantly changed. This study demonstrated that dry processing had a positive effect on some of the functional properties (water-holding, oil-holding, and emulsifying properties) of coconut isolate proteins, which could broaden the application of copra meal proteins in the food and beverage industry.

## Figures and Tables

**Table 1:** Proximate composition of defatted desiccated coconut, defatted copra meal, DCP, and CMP.

	Defatted Desiccated Coconut	Defatted Copra Meal	DCP	CMP
Moisture (%)	5.32 ± 0.17	9.11 ± 0.34	7.26 ± 0.31	7.85 ± 0.26
Carbohydrate (%)	37.89 ± 1.23	30.91 ± 0.81	18.17 ± 0.11	13.94 ± 0.27
Protein (%)	43.50 ± 1.56	47.37 ± 1.07	56.28 ± 0.18	58.11 ± 2.14
Fat (%)	5.15 ± 0.15	3.24 ± 0.86	3.95 ± 0.34	1.99 ± 0.03
Ash (%)	3.13 ± 0.13	5.79 ± 0.37	3.04 ± 0.19	6.57 ± 1.38

**Table 2:** Amino acid composition of protein fraction compared with soy isolate protein (SIP) ^a^ (mg/g protein).

Amino Acid ^b^	DCP	CMP	SIP ^a^	WHO/FAO ^c^	
				Child	Adult
Essential amino acid					
Thr	37.53	36.83	30.72	34.00	9.00
Val	70.24	66.85	47.87	35.00	13.00
Met	29.08	26.73	15.41		
Ile	48.77	44.61	60.78	28.00	13.00
Leu	85.57	82.98	63.39	66.00	19.00
Phe	55.72	56.97	45.15		
Lys	28.89	33.18	68.92	58.00	5.00
His	22.87	23.95	22.14	19.00	16.00
Total essential amino acid	378.67	372.10	326.50		
Non-essential amino acid					
Asp	93.84	95.59	121.68		
Ser	46.25	45.55	55.79		
Glu	181.61	197.80	180.94		
Gly	49.69	49.56	42.44		
Ala	51.63	50.95	43.74		
Cys	4.31	4.18	18.89		
Arg	119.97	113.32	83.03		
Tyr	35.34	31.50	34.52		
Pro	38.70	39.45	64.58		
EAA/TAA (%)	37.87	37.21	35.44		
Amino acids with different characterization ^d^					
Basic	171.73	170.45	174.10		
Acidic	275.44	293.39	302.62		
Charged polar	447.17	463.84	476.72		
Hydrophobic	379.71	368.54	340.93		
Sulfur-containing	33.39	30.91	34.30		

^a^ Data of soy protein isolate were reported by Yu et al. [11]. ^b^ The: Threonine; Val: Valine; Met: Methionine; Ile: Isoleucine; Leu: Leucine; Phe: Phenylalanine; Lys: Lysine; His: Histidine; Asp: Aspartic acid; Ser: Serine; Glu: Glutamic acid; Gly: Glycine; Ala: Alanine; Cys: Cysteine; Arg: Arginine; Tyr: Tyrosine; Pro: Proline. ^c^ Essential amino acid [FAO/WHO, 2007] required by a child or adult aged 3–5. ^d^ Basic amino acids: Lys, Arg, His; acidic amino acids: Asp, Glu; charged polar amino acids: basic and acidic amino acids; hydrophobic amino acids: Ala, Ile, Leu, Met, Phe, Val, Pro; sulfur-containing amino acids: Cys, Met.

**Table 3:** Relative content of the secondary structural features of SIP, DCP, and CMP. SIP: soy isolate protein; DCP: isolate protein from desiccated coconut; CMP: isolate protein from copra meal.

Sample	Secondary Structures (%)			
	β-Sheet	α-Helix	β-Turn	Random
SIP	33.85	10.94	31.38	23.82
DCP	36.43	11.62	26.19	25.76
CMP	31.39	11.7	31.72	25.18

## Data Availability

The data presented in this study are available on request from the corresponding author. The data are not uploaded in publicly accessible databases.

## References

[1] Zhang Y.F., Kan J.T., Liu X.Y., Song F., Zhu K.X., Li N. (2024). Chemical components, nutritional value, volatile organic compounds and biological activities in vitro of coconut (*Cocos nucifera* L.) water with different maturities. Foods.

[2] Saraiva A., Carrascosa C., Ramos F., Raheem D., Lopes M., Raposo A. (2023). Coconut sugar: Chemical analysis and nutritional profile; Health impacts; Safety and quality control; Food industry applications. Int. J. Environ. Res. Public Health.

[3] Divya P.M., Roopa B.S., Manusha C., Balannara P. (2022). A concise review on oil extraction methods, nutritional and therapeutic role of coconut products. J. Food Sci. Tech. Mys..

[4] Wu J.W., Tang Y.J., Chen W.X., Chen H.M., Zhong Q.P., Pei J.F. (2023). Mechanism for improving coconut milk emulsions viscosity by modifying coconut protein structure and coconut milk properties with monosodium glutamate. Int. J. Biol. Macromol..

[5] Chambal B., Bergenståhl B., Dejmek P. (2012). Edible proteins from coconut milk press cake; one step alkaline extraction and characterization by electrophoresis and mass spectrometry. Food Res. Int..

[6] Xiong J., Wu H.Y., Ye J. (2017). Variation of structures of ingredients of desiccated coconut during hydrolysis by hydrochloric acid at low temperature. Food Sci. Technol..

[7] Martínez-Padilla L.P., Hernández-Rojas F.S., Sosa-Herrera M.G., Juliano P. (2022). Novel application of ultrasound and microwave-assisted methods for aqueous extraction of coconut oil and proteins. J. Food Sci. Technol..

[8] Kumalasari I.D., Santosa I., Sulistiawati E. (2020). Coconut Oil Production with Various Roasting Temperatures and Dried Grated Coconut as a By-Product. IOP Conf. Ser. Earth Environ. Sci..

[9] Zheng Y.J., Li Y., Zhang Y.L., Zhao S.L. (2016). Purification, characterization and synthesis of antioxidant peptides from enzymatic hydrolysates of coconut (*Cocos nucifera* L.) cake protein isolates. Rsc. Adv..

[10] Wu J.W., Tang Y.J., Zhang M., Chen W.X., Chen H.H., Zhong Q.P. (2024). Mechanism for improving the in vitro digestive properties of coconut milk by modifying the structure and properties of coconut proteins with monosodium glutamate. Food Res. Int..

[11] Yu X.Y., Zou Y., Zheng Q.W., Lu F.X., Li D.H., Guo L.Q., Lin J.F. (2021). Physicochemical, functional and structural properties of the major protein fractions extracted from *Cordyceps militaris* fruit body. Food Res. Int..

[12] Rodsamran P., Sothornvit R. (2018). Physicochemical and functional properties of protein concentrate from by-product of coconut processing. Food Chem..

[13] Patil U., Benjakul S. (2017). Characteristics of albumin and globulin from coconut meat and their role in emulsion stability without and with proteolysis. Food Hydrocoll..

[14] Deng Y.J., Huang L.X., Zhang C.H., Xie P.J. (2019). Chinese quince seed proteins: Sequential extraction processing and fraction characterization. J. Food Sci. Technol..

[15] Zhang Y.X., Liu J.H., Lv J.R., Li Q.Y., Oh D.H., Fu X. (2023). Ultrasound-assisted glycation on ovalbumin fibrosis: A novel, efficient immobilization for lipase. Food Biosci..

[16] Pi J.Z., Wang J., Lv J.R., Jin Y.G., Oh D.H., Fu X. (2023). Modification of ovalbumin by the enzymatic method: Consequences for foaming characteristics of fibrils. Food Hydrocoll..

[17] Naik A., Raghavendra S.N., Raghavarao K.S.M.S. (2012). Production of coconut protein powder from coconut wet processing waste and its characterization. Appl. Biochem. Biotechnol..

[18] Pillai M.G., Thampi B.S., Menon V.P., Leelamma S. (1999). Influence of dietary fiber from coconut kernel (*Cocos nucifera*) on the 1,2-dimethylhydrazine-induced lipid peroxidation in rats. J. Nutr. Biochem..

[19] Fathollahy I., Farmani J., Kasaai M.R., Hamishehkar H. (2021). Characteristics and functional properties of Persian lime (*Citrus latifolia*) seed protein isolate and enzymatic hydrolysates. LWT-Food Sci. Technol..

[20] Sun Y., Chen H., Chen W., Zhong Q., Zhang M., Shen Y. (2022). Effects of ultrasound combined with preheating treatment to improve the thermal stability of coconut milk by modifying the physicochemical properties of coconut protein. Foods.

[21] Xu Y., Yang Y., Ma C.M., Bian X., Liu X.F., Wang Y., Chen F.l., Wang B., Zhang G., Zhang N. (2023). Characterization of the structure, antioxidant activity and hypoglycemic activity of soy (*Glycine max* L.) protein hydrolysates. Food Res. Int..

[22] Liu F.F., Li Y.Q., Wang C.Y., Liang Y., Zhao X.Z., He J.X., Mo H.Z. (2022). Physicochemical, functional and antioxidant properties of mung bean protein enzymatic hydrolysates. Food Chem..

[23] Ai M.M., Tang T., Zhou L.D., Ling Z.T., Guo S.G., Jiang A.M. (2019). Effects of different proteases on the emulsifying capacity, rheological and structure characteristics of preserved egg white hydrolysates. Food Hydrocoll..

[24] Mo X.Q., Wang D.H., Sun X.S. (2011). Physicochemical properties of β and α′α subunits isolated from soybean β-conglycinin. J. Agric. Food Chem..

[25] Azargohar R., Nanda S., Rao B.V.S.K., Dalai A.K. (2013). Slow pyrolysis of deoiled canola meal: Product yields and characterization. Energy Fuels.

[26] Li Y., Kong B., Xia X. (2013). Structural changes of the myofibrillar proteins in common carp (*Cyprinus carpio*) muscle exposed to a hydroxyl radical-generating system. Process Biochem..

[27] Wang K., Zhao X., Gantumur M.A., Li J., Huang Y., Sukhbaatar N. (2023). Extrusion of casein and whey protein isolate enhances anti-hardening and performance in high-protein nutrition bars. Food Chem. X.

[28] Sante-Lhoutellier V., Aubry L., Gatellier P. (2007). Effect of oxidation on in vitro digestibility of skeletal muscle myofibrillar proteins. J. Agric. Food Chem..

[29] Jiang S., Ding J., Andrade J., Rababah T.M., Almajwal A., Abulmeaty M.M., Feng H. (2017). Modifying the physicochemical properties of pea protein by phshifting and ultrasound combined treatments. Ultrason. Sonochem..

[30] Lyu S., Chen M., Wang Y., Zhang D., Zhao S., Liu J. (2023). Foaming properties of egg white proteins improved by enzymatic hydrolysis: The changes in structure and physicochemical properties. Food Hydrocoll..

[31] Boye J., Zare F., Pletch A. (2010). Pulse proteins: Processing, characterization, functional properties and applications in food and feed. Food Res. Int..

[32] Du H., Lin Y., Stanton C., Daniloski D., Zannini E., Ross R.P. (2023). Characterization and functional properties of pH- and heated time-induced aggregates from red lentil protein. Food Struc..

[33] Delahaije R.J.B.M., Gruppen H., Giuseppin M.L.F., Wierenga P.A. (2014). Quantitative description of the parameters affecting the adsorption behaviour of globular proteins. Colloid. Surface B.

[34] Tian Y., Pi J., Lv J., Chen Y., Ma M., Fu X. (2024). The impact of ultrasound treatment combined with flaxseed gum on the foaming properties of egg white. Food Hydrocoll..

[35] Kang Z.L., Bai R., Lu F., Zhang T., Gao Z.S., Zhao S.M. (2022). Effects of high pressure homogenization on the solubility, foaming, and gel properties of soy 11S globulin. Food Hydrocoll..

[36] Ma Y.G., Zhang J., He J.M., Xu Y.J., Guo X.B. (2023). Effects of high-pressure homogenization on the physicochemical, foaming, and emulsifying properties of chickpea protein. Food Res. Int..

